# Clinical characteristics and prognosis of patients with idiopathic membranous nephropathy with kidney tubulointerstitial damage

**DOI:** 10.1080/0886022X.2023.2205951

**Published:** 2023-04-26

**Authors:** Mengyao Sun, Ping Li, Jianwei Dong, Zhuo Li, Chaofan Li, Shasha Zhang, Bing Chen

**Affiliations:** a Department of Nephrology, Shandong Provincial Hospital Affiliated to Shandong First Medical University, Jinan, Shandong, China; b Department of Nephrology and Rheumatology, Affiliated Hospital of Shandong Medical College, Linyi, Shandong, China; c Department of Thoracic surgery, The people’s Hospital of Rongcheng, Rongcheng, Shandong, China; d Department of Nephrology, Shandong Provincial Hospital, Cheeloo College of Medicine, Shandong University, Jinan, Shandong, China

**Keywords:** Idiopathic membranous nephropathy, tubulointerstitial injury, tacrolimus, cyclosporine, cyclophosphamide

## Abstract

**Background:**

To investigate the clinical and kidney pathological features and prognosis of idiopathic membranous nephropathy (IMN) with kidney tubulointerstitial damage (TID).

**Methods:**

Based on the presence or absence of kidney TID by kidney biopsy, 300 patients diagnosed with IMN were categorized into non-TID (TID−) and tubulointerstitial injury (TID+) groups. The clinical and pathological data were analyzed retrospectively. All patients were followed up for 6–24 months after treatment with glucocorticoids (GCs) combined with cyclophosphamide or GCs combined with calcineurin inhibitors (CNIs) to observe treatment effects on patient prognosis.

**Results:**

The patients in the TID + group were older and more likely to be male. The 24-h urine protein, blood urea nitrogen, serum creatinine, cystatin C, β2-microglobulin, and antiphospholipase *A2 receptor* antibody levels were higher than those in the TID − group and the pathological manifestations were more severe. After 1 year of follow-up, the overall response rate (complete response + partial response) in the TID + group was lower (66.67% vs. 80.89%, *p* = .022) than in the other. After combined GC and CNI therapy, the complete remission rate in the TID + group was significantly lower than that in the TID − group (13.79% vs. 35.46%, *p* = .022). The 24-h urine protein level was an independent risk factor for worsening kidney condition *(p* = .038).

**Conclusion:**

Patients with IMN with TID have more severe clinical manifestations and pathological damage and lower remission rates. IMN with TID is a risk factor for worsening kidney condition; however, it is not an independent risk factor.

## Introduction

Membranous nephropathy (MN) is China’s most common type of adult nephrotic syndrome (NS) and is categorized into idiopathic membranous nephropathy (IMN) and secondary membranous nephropathy (SMN) based on its cause. Recently, IMN incidence has increased annually, accounting for 20% of primary glomerular diseases and 70%–80% of MN, and it is the second or third major cause of end-stage kidney disease (ESKD) [1,2]. IMN is a glomerular disease whose pathogenesis has not been fully elucidated. It is an antibody-mediated autoimmune kidney disease. Antigens deposited under the epithelium of the glomerular basement membrane form immune complexes with antibodies in the blood, activating complements and causing diffuse thickening of the basement membrane, causing severe damage to the glomerular filtration membrane and resulting in massive proteinuria. Approximately one-third of patients with IMN can spontaneously achieve remission after maintaining the course for several years, and two-thirds of patients with IMN will continue to deteriorate and gradually progress to ESKD even with active treatment and have long-term persistent proteinuria [3].

Previous studies have shown that factors including age, sex, proteinuria, serum creatinine (Scr) level, blood pressure, anti-phospholipase *A2 receptor* (anti-PLA2R) antibodies, and inappropriate immunosuppressive therapy use are IMN prognosis-related [4–5]. Additionally, Chen et al. found that tubulointerstitial damage (TID) was an independent risk factor for progression to ESKD in patients with IMN [6]. However, few studies are available on the role of TID in IMN progression and prognosis, and the relationship between TID and IMN prognosis has yet to be evaluated. To further explore the clinical and pathological characteristics of patients with IMN with TID and their prognosis under different treatment plans, we objectively analyzed and judged the factors influencing IMN prognosis, which has important clinical significance for evaluating the prognosis of patients with IMN and guiding the selection of appropriate individualized treatment plans.

Here, through the pathological examination of kidney biopsy, we found that approximately one-fifth of the patients with IMN had TID and further performed a comparative analysis of the clinical and pathological features of patients with IMN with and without tubulointerstitial injury. In this study, we compared the difference in prognosis between the two groups of patients after using different treatment plans, made recommendations for treating MN with TID, and analyzed the risk factors affecting IMN.

## Methods

### Study population

Overall, 300 patients diagnosed with IMN by kidney biopsy between January 2015 and December 2019 at Shandong Provincial Hospital affiliated with Shandong First Medical University were retrospectively reviewed. The Medical Ethics Committee of the Shandong Provincial Hospital affiliated with Shandong First Medical University reviewed and approved this study (ethics number: 2019-105); the study strictly followed the medical ethics standards. All patients fulfilled the NS criteria (24-h urine protein > 3.5 g/24 h, serum albumin < 30 g/L) and had normal kidney function, and none received regular immunosuppressive therapy. The exclusion criteria included the following: (1) incomplete clinical data; (2) suspicious history of nephrotoxic drugs; (3) being positive for tumor markers or having undetermined imaging or gastrointestinal endoscopy evidence of tumor; (4) antinuclear antibody >1:320; (5) electron-dense deposition observed in the mesangial area under electron microscopy; (6) infectious, autoimmune, and malignant diseases and SMN caused by other factors; and (7) incomplete follow-up data.

### Clinical and laboratory parameters

General clinical parameters, including age, sex, and systolic and diastolic blood pressure at admission, were collected. Laboratory parameters included 24-h urine protein, hemoglobin, leukocytes, platelets, alanine aminotransferase (normal range: 9–50 u/L), aspartate aminotransferase (normal range: 15–40 u/L), albumin (normal range: 40–55 g/L), total protein (normal range: 65–85 g/L), blood glucose (normal range: 3.9–6.3 mmol/L), serum β2-microglobulin (normal range: 1.0–3.0 mg/L), blood urea nitrogen (BUN, normal range: 2.8–7.14 mmol/L), blood calcium (normal range: 2.2–2.7 mmol/L), cystatin C (normal range: 0.63–1.25 mg/L) and Scr (normal range: 40–135µmol/L). Immunological indicators included serum IgG (normal range: 7–16 g/L), IG4 (normal range: 30–1350mg/L), IgA (normal range: 0.7–4.0g/L), and IgM (normal range: 0.4–2.30g/L) levels; serum complement C3 (normal range: 0.8–1.8g/L) and C4 (normal range: 0.1–0.4g/L) levels. Serum PLA2R antibody was detected using an enzyme-linked immunosorbent assay. A modified formula was used to calculate the estimated glomerular filtration rate (eGFR, normal range: >90 mL/min/1.73m^2^) as follows: eGFR [mL/min/1.73m^2^] = 186 × [Scr (μmol/L)/88.4] − 1.154 × age − 0.203 (×0.742 if female).

### Pathological data

**Figure 1.**
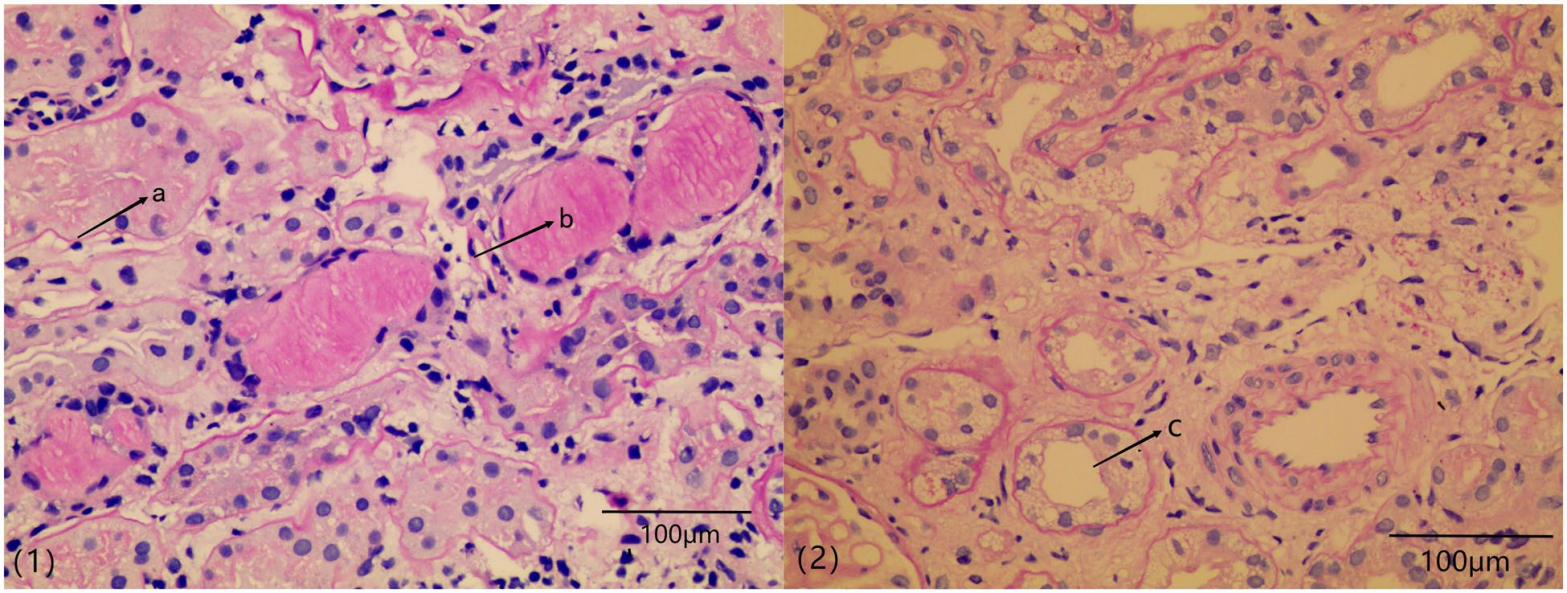
Renal biopsy specimen with acute and chronic tubulointerstitial lesions. (1) shows an acute tubulointerstitial lesion, where a indicates tubular epithelial edema and b indicates tubular epithelial necrosis; (2) shows a chronic tubulointerstitial lesion, where c indicates interstitial fibrosis. (1) and (2) are both Periodic acid Schiff (PAS) staining ×400.

We performed light microscopy, immunofluorescence, and electron microscopy of the kidney puncture tissues. Kidney biopsies from each patient included at least 10 glomeruli for histopathological evaluation. MN was categorized into four stages. When two phases were observed, the higher phase was selected. Spherical sclerosis was defined as the hardening of a small sphere that could be observed under a light microscope. Glomerular mesangial hyperplasia was defined as ≥ 4 cells in the mesangial region under a light microscope. Inflammatory cell infiltration was defined as the infiltration of neutrophils. Small vessel lesions included arterial intimal thickening, edema, sclerosis, and hyaline or mucinous degeneration. Acute tubulointerstitial lesions were defined as tubular epithelial edema or necrosis (severe epithelial vacuolation and granular degeneration, disintegration, etc.) of at least 10%, as shown Figure 1(1). Chronic tubulointerstitial lesions were defined as tubular atrophy (reduced epithelial cell volume, thickened basement membrane, lumen narrowing, etc.) and interstitial fibrosis (interstitial collagen hyperplasia) of at least 10%, as shown Figure 1(2). Crescent lesions were the presence of a cellular or fibrous crescent in one glomerulus. Hyperplasia endothelialis was defined as the presence of proliferative endothelial lesions. Balloon adhesion was defined as the partial or complete fusion of glomerular capillaries with the basement membrane of the kidney vesicle. The capillaries were pulled to the side of the basement membrane of the kidney vesicle. Focal segmental glomerulersclerosis (FSGS) lesions were characterized by a focal and segmental distribution of glomerular lesions under a light microscope, with a small increase in the mesangial matrix and balloon adhesion as the primary manifestations.

### Group standard

According to the Ehrenreich–Chrug MN staging standard [7], and simultaneously, based on the proportion (%) of kidney tubular atrophy and kidney interstitial fibrosis, semiquantitative scoring of kidney tubule-interstitial lesions was performed, including the scoring standards for various interstitial lesions: 0 points, no interstitial lesions; one point, interstitial involvement <10%; two points, 10% ≤ interstitial involvement ≤25%; three points, 25% < interstitial involvement range ≤50%; and four points, interstitial involvement range >50%. Here, TID was a score of > 2. Patients with TID were categorized into the nontubulointerstitial (TID−, *N* = 246) and tubulointerstitial (TID+, *N* = 54) injury groups.

### Treatment and follow-up records

With the training of the hospital’s nephrology department, all doctors treated all patients according to specified guidelines and principles, unifying the dosing schedule and pattern of treatment protocols. The treatment regimens of 300 patients with IMN primarily included glucocorticoids (GC) and immunosuppressants (cyclophosphamide [CYC], calcineurin inhibitors [CNI], cyclosporine A [CsA], tacrolimus [TAC]), supportive treatments, including angiotensin-converting enzyme inhibitors (ACEIs) or angiotensin type 1 receptor blockers (ARBs), and symptomatic treatments, including diuresis and lipid-lowering. ACEIs and ARBs were used to control blood pressure to below 140/90 mmHg. When the albumin level was ˂25 g/L, prophylactic anticoagulation was administered to prevent thrombosis. According to the IMN treatment proposed by the 2012 KDIGO guidelines [8], CYC treatment was preferred for those who satisfied the initial treatment criteria and had no contraindications to CYC use. The application of CNI was contraindicated in the following cases: (1) for the use of CYC (e.g., allergy to CYC and its metabolites, severe bone marrow suppression, cystitis, urinary tract obstruction, acute infections, etc.); (2) when it was difficult to regularly inject CYC in the hospital due to inconvenient access to medical care; (3) when high doses of glucocorticoids were not tolerated; (4) when patients had side effects that would occur with high doses of glucocorticoids (hyperglycemia, diabetes mellitus, etc.). GC for all patients was prednisone. The treatment regimen of GC combined with CYC was as follows: CYC 10–15 mg/(kg·M) (intravenous) once a month and monthly monitoring of leukocyte count, 24-h urine protein, Scr, albumin, and other items. All patients were given an adequate dose of prednisone acetate 1 mg/(kg·d). The treatment lasted for at least 6 months. The treatment regimen of GC combined with TAC was as follows: TAC was 0.05–0.075 mg/(kg·d) (12 h apart) and prednisone was 0.3–0.5 mg/(kg·d) for 6–12 months. The dose of prednisone was gradually reduced after 8 weeks of treatment by 5 mg every 2 weeks to 10 mg/d for maintenance [8]. The regimen of GC combined with CsA was maintained at a CsA dose of 3.5–5.0 mg/(kg·d) (orally once every 12 h) and prednisone 0.3–0.5 mg/(kg·d). The treatment lasted for at least 6 months. In all three of the above treatment regimens, GC was gradually reduced after 8 weeks of treatment by 5 mg every 2 weeks to 10 mg/d for maintenance [8]. Both CsA and TAC are CNIs. If a complete or partial response was achieved after 6 months of CNI treatment, the dose was gradually reduced to 50% of the initial dose after 4–8 weeks and maintained for at least 12 months. The blood concentrations of CNI and TAC were monitored regularly and maintained at 5–10 ng/ml. The CsA concentration was maintained at 100–200 ng/ml. The complete response (CR), partial response (PR), and overall response (CR + PR) rates in the TID − and TID + groups were compared and analyzed.

Here, only the first immunotherapy regimen’s results were followed up; the follow-up was terminated at the end of the first regimen. Patients were required to receive standardized treatment for at least 6 months. Patients with early protocol changes or insufficient follow-up were excluded. All patients were followed up for over 6 months, with a median follow-up of 30.00 months. The primary and secondary endpoints were CR and PR after 1 year of treatment and worse kidney condition, respectively.

### Efficacy judgment and prognosis

According to the response criteria for IMN treatment, the clinical effect of treatment was assessed based on changes in 24-h urine protein and Scr levels. The CR rate was defined as urinary protein < 0.3 g/24 h or urinary protein/creatinine < 300 mg/g, normal kidney function, albumin > 35 g/L, and stable renal function. PR rate was defined as 24-h urine protein > 0.3 g/24 h; however, < 3.5 g/24 h or urinary protein/creatinine at 300–3500 mg/g or 24-h urine protein decreased 50% from baseline, and kidney function was normal. We defined worse kidney condition as a doubling of baseline Scr levels that persisted for more than 3 months after follow-up treatment. Recurrence was defined as the reachievement of the NS criteria 1 month after CR or PR. Serious complications included clinical death, severe lung infection, necrosis of the femoral head, and other diseases.

### Statistical analyses

All data were analyzed using IBM SPSS Statistics for Windows, version 22 (IBM Corp., Armonk, NY, USA). For measurement data, the Shapiro–Wilk test was used to determine whether the data were normally distributed. Normally distributed data were expressed as mean ± standard deviation (SD) and differences were analyzed by *t*-test; non-normally distributed data were expressed as median (lower and upper quartiles) and analyzed by nonparametric test. Count data were presented as an example (rate) and analyzed using the chi-square test (*χ*^2^). Spearman’s rank correlation coefficient was used to analyze the correlation between the parameters of the nonparametric distribution. Cox regression analysis was used to exclude variables and independent risk factors related to worse kidney conditions and evaluate the relationship between covariables and patient and kidney survival. Statistical significance was set at *p* < .05.

## Results

### General clinical data

Among 300 patients with IMN, 246 (82.00%) and 54 (18.00%) were in the TID − and TID + groups, respectively. Baseline clinical data prior to treatment were retrospectively analyzed in both groups. The patients’ age in the TID + group was older than that in the TID − group (52.00 years vs. 47.00 years, *p* = .018), and there were more male patients in the TID + group than in the TID − group (77.78% vs. 63.01%, *p* = .038). Patients in the TID + group had significantly higher 24-h urine protein levels than patients in the TID − group (6.85 g/24 h vs. 5.36 g/24 h, *p* = .015). BUN (5.40 vs. 4.80 µmol/L, *p* = .010), Scr (78.00 vs. 76.00 µmol/L, *p* < .001), Cystatin C (1.05 vs. 0.91 mg/L, *p* < .001), and β2-microglobulin levels (2.68 vs. 2.09 mg/L, *p* < .001) were higher than those in the TID − group. The anti-PLA2R antibody level in the TID + group was also significantly higher than that in the TID − group (136.36 vs. 65.61 RU/ml, *p* = .001). Table 1 provides further details.

**Table 1.** Comparison of basic clinical features and laboratory tests between the two groups.

	IMN (*N* = 300)	TID − group (*N* = 246)	TID + group (*N* = 54)	*p* value
Age (year)	48.00 (36.00,56.00)	47.00 (36.00,54.25)	52.00 (38.75,58.00)	.018*
Male (%, *n*)	65.67 (197)	63.01 (155)	77.78 (42)	.038*
SBP (mmHg)	136.00 (122.00,150.00)	135.50 (122.00,150.00)	137.00 (122.00,151.25)	.537
DBP (mmHg)	87.00 (77.00,96.00)	86.50 (77.00,96.00)	87.00 (78.75,95.25)	.847
Urinary protein (g/24 h)	5.60 (4.18,7.90)	5.36 (4.05,7.49)	6.85 (4.72,8.96)	.015*
Hemoglobin (g/L)	140.00 (127.00,152.00)	141.00 (127.00,152.00)	136.50 (122.75,149.25)	.170
WBC (×10^9^/L)	6.44 (5.28,7.87)	6.44 (5.23,7.99)	6.31 (5.44,7.34)	.778
Platelets (×10^9^/L)	260.00 (225.00,305.00)	262.00 (226.50,309.00)	254.00 (222.00,304.25)	.530
AST (µ/L)	21.00 (18.00,26.00)	21.00 (17.00,25.00)	22.00 (18.00,27.50)	.176
ALT (µ/L)	18.00 (14.00,28.00)	19.00 (14.00,28.00)	17.00 (13.50,22.50)	.222
Superoxide dismutase (µ/mL)	111.05 (96.28,125.08)	111.75 (98.25,125.53)	104.80 (84.90,121.68)	.165
Serum total protein (g/L)	47.00 (42.00,51.80)	47.40 (42.35,51.70)	44.60 (40.80,52.10)	.116
Albumin (g/L)	23.55 (20.40,27.00)	23.80 (20.65,27.05)	22.70 (19.60,26.20)	.150
Serum creatinine (µmol/L)	76.20 (72.00,79.00)	76.00 (71.00,78.75)	78.00 (73.85,84.08)	<.001*
eGFR (ml/min/1.73m^2^)	95.27 (80.24,106.17)	95.59 (81.76,108.41)	92.07 (77.12,104.53)	.075
BUN (mmol/L)	4.95 (3.90,6.33)	4.80 (3.90,6.10)	5.40 (4.48,6.73)	.010*
Cystatin C (mg/L)	0.93 (0.82,1.09)	0.91 (0.80,1.05)	1.05 (0.93,1.21)	<.001*
β2-microglobulin (mg/L)	2.20 (1.80,2.70)	2.09 (1.74,2.59)	2.68 (2.36,3.49)	<.001*
RBP (mg/L)	54.10 (45.30,65.80)	52.80 (45.15,65.75)	57.20 (45.50,70.45)	.242
Blood calcium (mmol/L)	2.09 (2.00,2.21)	2.08 (2.00,2.22)	2.13 (1.99,2.21)	.787
CHOL (mmol/L)	8.53 (7.03,10.26)	8.46 (6.95,10.12)	9.30 (7.18,10.95)	.288
HDL-C (mmol/L)	1.48 (1.26,1.88)	1.52 (1.26,1.90)	1.41 (1.25,1.72)	.113
LDL-C (mmol/L)	5.23 (4.02,6.71)	5.18 (3.98,6.65)	5.67 (4.32,7.06)	.337
Blood glucose (mmol/L)	5.05 (4.70,5.55)	5.05 (4.70,5.57)	5.04 (4.76,5.50)	.935
Anti-PLA2R antibody (RU/ml)	77.96 (16.29,216.13)	65.61 (14.54,182.64)	136.36 (64.83,308.89)	.001*
IgG4 (mg/L)	302.00 (174.50,449.25)	302.00 (172.00,451.00)	329.00 (175.00,440.00)	.869
IgG (g/L)	5.47 (4.08,6.86)	5.55 (4.19,7.06)	4.77 (3.50,6.29)	.057
IgM (g/L)	1.01 (0.77,1.43)	1.04 (0.79,1.45)	0.97 (0.62,1.40)	.152
IgA (g/L)	2.21 (1.76,2.79)	2.22 (1.82,2.85)	2.06 (1.50,2.62)	.053
Complement C3 (g/L)	1.20 (1.07,1.34)	1.20 (1.08,1.31)	1.22 (1.06,1.44)	.930
Complement C4 (g/L)	0.30 (0.25,0.35)	0.30 (0.25,0.35)	0.30 (0.25,0.34)	.980
Complement C1q (mg/L)	216.00 (189.50,242.00)	214.00 (189.75,241.12)	224.00 (185.00,250.00)	.491

WBC: white blood cell; AST: glutamic oxaloacetic transaminase; ALT: glutamic pyruvic transaminase; BUN: blood urea nitrogen; eGFR: estimated Glomerular filtration rate; RBP: retinol binding protein; CHOL: total cholesterol; HDL-C: high-density lipoprotein; LDL-C: low-density lipoprotein; IgG4: immunoglobulin G4; IgG: immunoglobulin G; IgM: immunoglobulin M; IgA: immunoglobulin A; SBP: systolic blood pressure; DBP: diastolic blood pressure.

*Between the TID − group and the TID + group.

### Pathological data

**Figure 2.**
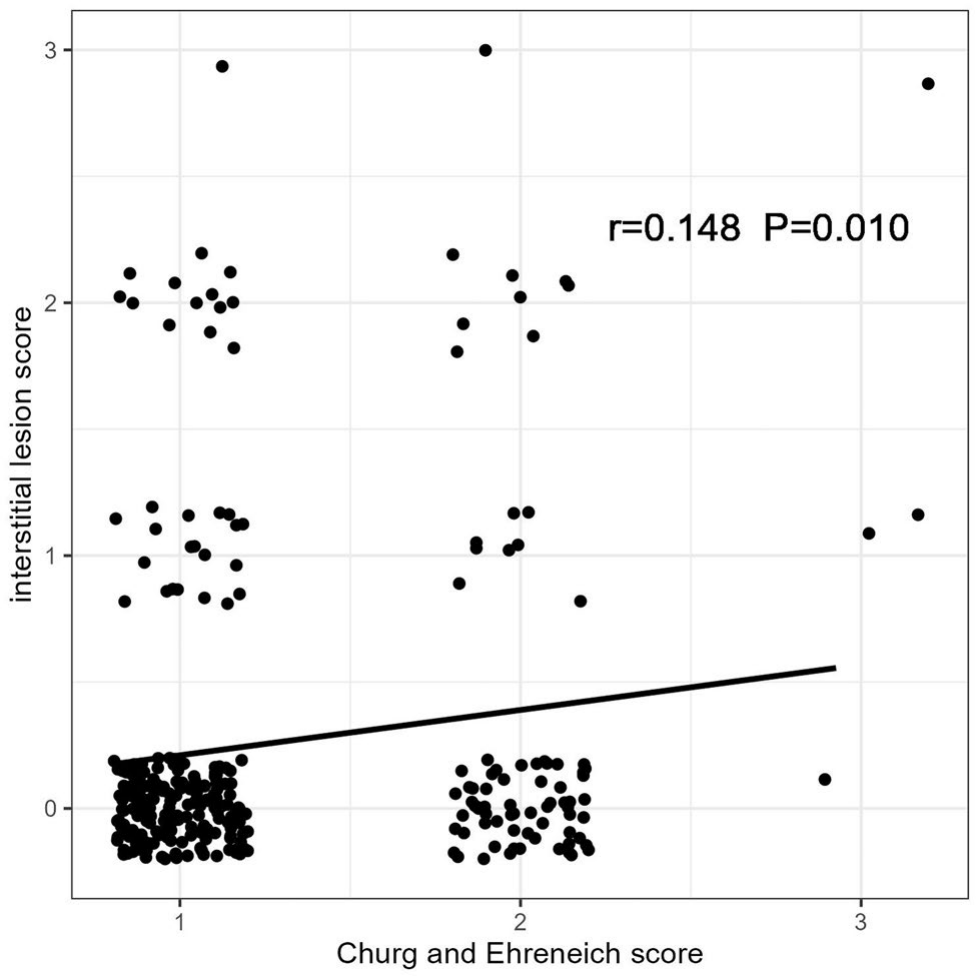
The correlation between Ehrenreich–Chrug scores and tubulointerstitial lesion score. Scatter plot in Spearman correlation analysis shows the correlation between the Ehrenreich–Chrug score and interstitial lesion score.

The pathological stages of patients with IMN in the TID − and TID + groups were primarily stages I and II from the pathological data analysis. The proportion of patients with stage III disease in the TID + group was higher than that in the TID − group (5.56% vs. 0.41%, *p* = .012). As shown in Figure 2, Spearman correlation analysis showed a positive correlation between Ehrenreich-Chrug scores and tubulointerstitial lesion score (*r* = 0.148, *p* = .010). The rates of spherical sclerosis (68.52% vs. 47.56%, *p* = .005), glomerular mesangial hyperplasia (68.52% vs. 39.02%, *p* < .001), inflammatory cell infiltration (48.15% vs. 14.23%, *p* < .001), small vessel lesions (55.56% vs. 33.74%, *p* = .003), balloon adhesion (51.85% vs. 4.47%, *p* < .001), acute tubulointerstitial lesions (96.30% vs. 17.07%, *p* < .001), and FSGS (55.56% vs. 17.07%, *p* < .001) were significantly higher in the TID + group than in the TID − group. Table 2 provides further details.

**Table 2.** Histopathological parameters of kidney biopsy.

	TID − group (*N* = 246)	TID + group (*N* = 54)	*P* value
Pathological stage (%, *n*)			
I	74.39 (183)	62.96 (34)	0.089
II	25.20 (62)	31.48 (17)	0.343
III	0.41 (1)	5.56 (3)	0.012*
IV	0.00 (0)	0.00 (0)	–
Pathological characteristics (%, *n*)			
Spherical sclerosis	47.56 (117)	68.52 (37)	0.005*
Glomerular mesangial hyperplasia	39.02 (96)	68.52 (37)	<0.001*
Inflammatory cell infiltration	14.23 (35)	48.15 (26)	<0.001*
Small vessel lesions	33.74 (83)	55.56 (30)	0.003*
Acute tubulointerstitial lesions	4.47 (11)	96.30 (52)	<0.001*
Crescents	2.44 (6)	5.56 (3)	0.438
Hyperplasia endothelialis	5.28 (13)	3.70 (2)	0.890
Balloon adhesion	17.07 (42)	51.85 (28)	<0.001*
FSGS	15.45 (38)	55.56 (30)	<0.001*

FSGS: focal segmental glomerulersclerosis.

*Between the TID − group and the TID + group.

### Treatment and outcomes

After 1 year of follow-up treatment in 300 patients with IMN, the TID + group CR + PR rate was significantly lower than that of the TID − group (66.67% vs. 80.89%, *p* = .022), and the TID + group CR rate was significantly lower than that of the TID − group (16.67% vs. 34.96%, *p* = .009). Among 130 patients with IMN treated with GC + CYC, the TID + group CR + PR rate was lower than that in the TID − group (60.00% vs. 78.10%, *p* = .062), and the TID + group CR rate was significantly lower than that of the TID − group (20.00% vs. 34.29%, *p* > .05). Among patients with IMN treated with GC + CNI, the TID + group CR + PR rate was lower than that in the TID − group (72.41% vs. 82.98%, *p* > .05), and the CR rate was significantly lower than that in the TID − group (13.79% vs. 35.46%, *p* = .022). This indicates that after 1 year of treatment, patients in the TID + group had more difficulty achieving remission than those in the TID − group, both in the GC + CYC and GC + CNI groups and particularly in the GC + CNI group. Table 3 provides further details.

**Table 3.** Comparison of remission rate between two groups after treatment.

Total	CR (%, *n*)	PR (%, *n*)	CR + PR (%, *n*)
IMN (*N* = 300)			
TID − group (*N* = 246)	34.96 (86)	45.93 (113)	80.89 (199)
TID + group (*N* = 54)	16.67 (9)	50.00 (27)	66.67 (36)
*p* value	.009*	.588	.022*
GC + CYC (*N* = 130)			
TID − group (*N* = 105)	34.29 (36)	43.81 (46)	78.10 (82)
TID + group (*N* = 25)	20.00 (5)	40.00 (10)	60.00 (15)
*p* value	.167	.730	.062
GC + CNI (*N* = 170)			
TID − group (*N* = 141)	35.46 (50)	47.52 (67)	82.98 (117)
TID + group (*N* = 29)	13.79 (4)	58.62 (17)	72.41 (21)
*p* value	.022*	.276	.185
GC + CsA (*N* = 81)			
TID − group (*N* = 67)	31.34 (21)	49.25 (33)	80.60 (54)
TID + group (*N* = 14)	14.29 (2)	57.14 (8)	71.43 (10)
*p* value	.336	.591	.685
GC + TAC (*N* = 89)			
TID − group (*N* = 74)	39.19 (29)	45.95 (34)	85.14 (63)
TID + group (*N* = 15)	13.33 (2)	60.00 (9)	73.33 (11)
*p* value	.055	.321	.462

CR: complete remission rate; PR: partial remission rate; CsA: cyclosporin; GC: glucocorticoids; TAC: tacrolimus; CYC: cyclophosphamide; CNI: calcineurin inhibitors.

*Between the TID − group and the TID + group.

After treatment, 24-h urine protein and Scr levels of patients with IMN in the TID − and TID + groups were significantly decreased, and albumin, BUN, and eGFR levels were significantly increased (all *p* < .001). Regarding the GC + CYC treatment regimen, 24-h urine protein and Scr levels of patients in the TID − and TID + groups were significantly decreased after treatment, and albumin and eGFR were significantly increased (all *p* < .001); Patients in both groups showed an increase in BUN levels after treatment, but it was not statistically significant (*p* > .05). Regarding the GC + CNI treatment regimen, 24-h urine protein and Scr levels of patients in the TID − and TID + groups were significantly decreased after treatment, and albumin and BUN levels were significantly increased (all *p* < .001); Patients in both groups had a decrease in eGFR levels after treatment, but there was no statistical significance in the TID + group (*p* > .05). Our results showed that the 24-h urine protein level was significantly higher in the TID + group than in the TID − group after treatment (1.70 g/24 h vs. 0.98 g/24 h, *p* = .007). The serum BUN level was significantly higher in the TID + group than in the TID − group after treatment (6.30 vs. 5.50 µmol/L, *p* = .002). The level of post-treatment to the pretreatment ratio of 24-h proteinuria was higher in the TID + group than in the TID − group (0.29 vs. 0.15, *p* = .035), while there was no significant difference in the response of the two groups before and after treatment for other indicators. Subgroup analysis showed that the 24-h urine protein level was significantly higher in the TID + group than in the TID − group in patients using GC combined with CNI (*p* < .05), and the Scr and BUN levels were also significantly higher in the TID + group than in the TID − group (*p* < .05). The eGFR level was significantly lower in the TID + group than in the TID − group. There were no significant differences in treatment effects (e.g., post-treatment to pretreatment ratio) between the two groups in either the GC + CYC or GC + CNI treatment regimens. Table 4 presents additional details.

**Table 4.** Laboratory parameters of IMN patients after treatment.

	GC + CYC (*N* = 130)			GC + CNI (*N* = 170)		
	TID− (*N* = 105)	TID+ (*N* = 25)	*p* value^a^	TID− (*N* = 141)	TID+ (*N* = 29)	*p* value^a^
Urine protein (g/24 h)	0.93 (0.21,2.70)	1.50 (0.63,4.32)	0.099	1.00 (0.19,2.58)	1.78 (0.50,4.20)	0.032
Decrease from pretreatment, % (*n*)	94	23	0.711	132	25	0.171
Ratio to pretreatment levels	0.17 (0.03,0.54)	0.27 (0.08,0.64)	0.318	0.14 (0.03,0.41)	0.31 (0.07,0.68)	0.054
*p* value^b^	<.001	<.001	–	<.001	<.001	–
Albumin (g/L)	37.00 (31.05,39.60)	35.60 (28.95,39.70)	0.475	38.00 (34.65,41.70)	37.90 (29.50,40.90)	0.337
Increase from pretreatment, % (*n*)	98	21	0.132	134	25	0.078
Ratio to pretreatment levels	1.49 (1.29,1.79)	1.71 (1.22,1.97)	0.140	1.60 (1.32,1.85)	1.44 (1.29,1.79)	0.339
*p* value^b^	<.001	<.001	–	<.001	<.001	–
Serum creatinine (µmol/L)	63.10 (56.00,70.55)	62.00 (52.62,78.50)	0.988	66.70 (56.70,76.35)	74.00 (65.25,89.95)	0.022
Decrease from pretreatment, % (*n*)	87	21	0.891	95	16	0.209
Ratio to pretreatment levels	0.82 (0.73,0.95)	0.78 (0.66,0.94)	0.127	0.90 (0.78,1.04)	0.97 (0.80,1.13)	0.217
*p* value^b^	<.001	.003	–	<.001	.509	–
eGFR (ml/min/1.73m^2^)	115.09 (103.66,131.05)	122.35 (94.23,143.90)	0.993	111.60 (92.14,126.12)	101.31 (74.32,115.10)	0.022
Increase from pretreatment, % (*n*)	87	21	0.891	94	16	0.238
Ratio to pretreatment levels	1.26 (1.07,1.43)	1.33 (1.07,1.62)	0.127	1.13 (0.96,1.34)	1.04 (0.87,1.29)	0.203
*p* value^b^	<.001	<.001	–	<.001	.247	–
BUN (mmol/L)	5.30 (4.60,6.20)	5.90 (4.85,7.80)	0.069	5.60 (4.90,6.90)	6.40 (5.50,8.80)	0.006
Decrease from pretreatment, % (*n*)	46	11	0.986	39	6	0.438
Ratio to pretreatment levels	1.05 (0.82,1.34)	1.06 (0.78,1.40)	0.938	1.22 (0.97,1.56)	1.32 (1.04,1.66)	0.309
*p* value^b^	.490	.686	–	<.001	<.001	–

GC: glucocorticoids; CYC: cyclophosphamide; CNI: calcineurin inhibitors; BUN: blood urea nitrogen; eGFR: estimated Glomerular filtration rate.

^a^Comparison between TID + group and TID − group.

^b^Comparison between pre and post treatment.

### Prognostic analysis

**Figure 3.**
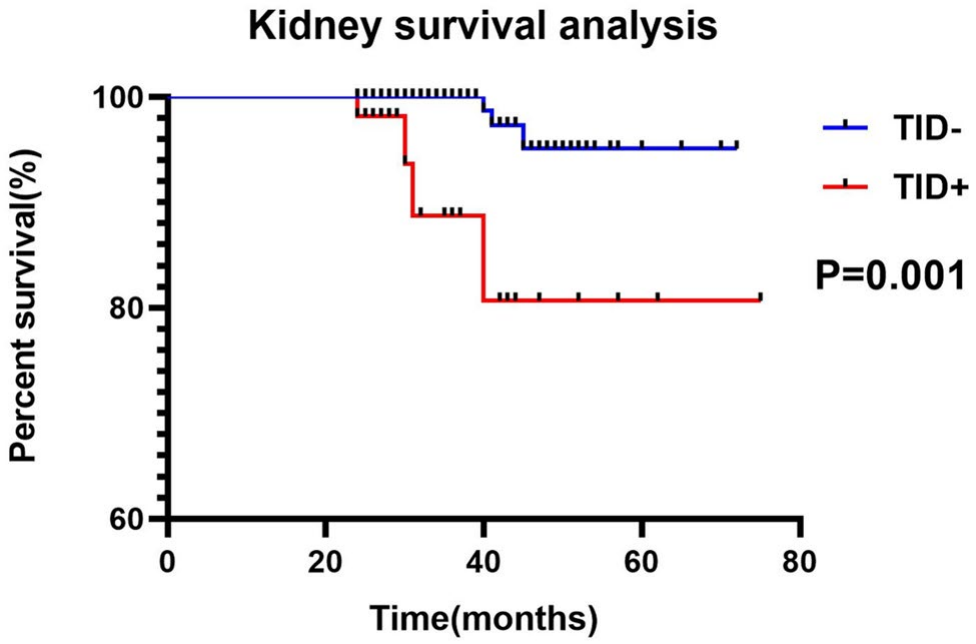
TID can predict kidney outcome in patients with IMN. See methods for the definition of worsening kidney condition. Kidney survival is depicted for patients with TID (TID+, red line, *N* = 54) and without TID (TID−, blue line, *N* = 246).

Among the common adverse reactions, the incidence of elevated blood glucose, infection, and hypertension in the TID + group was significantly higher than that in the TID − group. From the study to the secondary follow-up endpoint, seven patients had worse kidney conditions among the serious adverse reactions, with three and four cases in the TID − and TID + groups, respectively, and the difference was statistically significant (*p* = .021). Three cases occurred in the GC + CNI group, and excessive concentrations of CsA or TAC may have caused the worse kidney condition in these patients. Four cases occurred in the GC + CYC group; these patients were considered to be affected by IMN disease factors that led to a worse kidney condition. Table 5 presents additional details. Kaplan–Meier survival plots were used for log-rank tests, and the difference in kidney survival between the two groups was statistically significant (*χ*^2^ = 11.768, *p* = .001). The 3-year kidney survival rates were 88.8% and 100.0% in the TID + and TID − groups, respectively, as shown in Figure 3.

**Table 5.** The adverse reaction of the two groups was compared.

	TID − group (*N* = 246)	TID + group (*N* = 54)	*p* value
Common adverse reaction (%, *n*)			
Elevated blood glucose (*N* = 23)	7.32 (18)	9.26 (5)	.839
Infection (*N* = 21)	6.50 (16)	9.26 (5)	.672
Hypertension (*N* = 22)	7.72 (19)	5.56 (3)	.791
Cerebral infarction (*N* = 5)	1.63 (4)	1.85 (1)	.908
Joint pain (*N* = 19)	5.69 (14)	9.26 (5)	.505
Paroxysmal atrial flutter (*N* = 1)	0.41 (1)	0.00 (0)	.528
Herpes zoster (*N* = 8)	2.44 (6)	3.70 (2)	.955
Hair loss (*N* = 17)	6.10 (15)	3.70 (2)	.716
Gingival hyperplasia (*N* = 1)	0.00 (0)	1.85 (1)	.063
Severe adverse reaction (%, *n*)			
Severe pneumonia (*N* = 1)	0.00 (0)	1.85 (1)	.063
Necrosis of femoral head (*N* = 5)	0.81 (2)	5.56 (3)	.035*
worse kidney condition (*N* = 7)	1.22 (3)	7.41 (4)	.026*

*Between the TID − group and the TID + group.

Univariate COX regression analysis revealed that 24-h urine protein (*p* = .001), β2-microglobulin (*p* = .004), anti-PLA2R antibody (*p* = .023), chronic tubulointerstitial lesions (*p* = .021), FSGS (*p* = .049), and pathological stage III (*p* = .015) were risk factors for worse kidney conditions (Table 6). Multivariate COX regression analysis of statistically significant risk factors demonstrated that 24-h urine protein was an independent risk factor affecting worse kidney condition (*p* = .038) as presented in Table 7.

**Table 6.** Univariate Cox regression analysis of kidney function deterioration in IMN patients.

	B	SE	Wald	df	sig	Exp(B)
24-h urine protein	0.280	0.086	10.741	1	0.001	1.324 (1.119,1.565)
β2-microglobulin	0.822	0.287	8.221	1	0.004	2.276 (1.297,3.993)
Anti-PLA2R antibody	0.002	0.001	5.163	1	0.023	1.002 (1.000,1.003)
CTL	1.769	0.764	5.367	1	0.021	5.867 (1.313,26.216)
FSGS	1.505	0.764	3.883	1	0.049	4.505 (1.008,20.127)
Pathological stage			5.981	2	0.050	
Stage III	2.707	1.118	5.861	1	0.015	14.983 (1.674,134.080)

CTL: Chronic tubulointerstitial lesions; FSGS: focal segmental glomerulosclerosis.

**Table 7.** Multivariate Cox regression analysis of kidney function deterioration in IMN patients.

	B	SE	Wald	df	sig	Exp(B)
24-h urine protein	0.236	0.114	4.288	1	0.038	1.266 (1.013,1.582)
β2-microglobulin	0.159	0.447	0.127	1	0.722	1.173 (0.488,2.818)
Anti-PLA2R antibody	0.001	0.001	2.741	1	0.098	1.001 (1.000,1.003)
CTL	0.0571	1.168	0.239	1	0.625	1.770 (0.179,17.465)
FSGS	0.496	1.076	0.212	1	0.645	1.641 (0.199,13.534)
Pathological stage			1.005	2	0.605	
Stage III	1.359	1.396	0.948	1	0.330	3.894 (0.252,60.100)

CTL: Chronic tubulointerstitial lesions; FSGS: focal segmental glomerulosclerosis.

## Discussion

Recent studies have shown that the degree of TID is an essential factor leading to ESKD progression, affecting the long-term prognosis of patients with chronic kidney disease [9]. However, few reports are available on the clinicopathological features of IMN with TID. Studies have shown that hypertension, Scr, BUN, and urinary α-l microglobulin levels are associated with TID in IMN with clinical manifestations of NS [10]. This is consistent with this study’s results. Here, the levels of 24-h urine protein, BUN, Scr, Cystatin C, β2-microglobulin, and anti-PLA2R antibody in patients with IMN with TID were higher than those in patients without TID. Regarding pathology, we found that the pathological stages of patients with IMN were mainly in phases I and II, consistent with the relevant literature [11]. Additionally, the proportions of phases II and III in the TID + group were higher than those in the TID − group. The stage of Ehrenreich–Chrug MN was positively correlated with the tubulointerstitial lesion score (*r* = 0.148, *p* = .010). Stage III, IV, and patients with a high degree of tubulointerstitial injury were not numerous and the correlation was not strong, which needs to be confirmed in a large sample. We also observed that the proportions of spherical sclerosis, glomerular mesangial hyperplasia, inflammatory cell infiltration, small vessel lesions, acute tubulointerstitial lesions, balloon adhesion, and FSGS in the TID + group were higher than those in the TID − group. Gu et al. showed that kidney tubular atrophy, interstitial infiltration, and interstitial fibrosis are common and severe [12]. Some studies have found that the kidney lesions of patients with IMN with FSGS are significantly more serious, and interstitial fibrosis and vascular disease change markedly [13]. This finding is consistent with this study’s pathological results. Although glomerular mesangial hyperplasia exists in the pathological biopsy of some patients, SMN can be excluded in accordance with laboratory results and pathological manifestations, including blood anti-PLA2R antibody, antinuclear antibody, antidouble-stranded DNA antibody, decreased serum complement, or other positive infectious markers.

Pathological changes in IMN occur in the glomerulus, and the TID incidence rate is low. However, IMN with severe TID frequently is associated with serious conditions and has a poor prognosis in clinical practice [14]. Currently, most studies on IMN have focused on the glomerular injury. The impact of pathological manifestations of concomitant kidney tubular interstitial injury on the disease process and prognosis of IMN should be fully appreciated. Studies have demonstrated that CR is associated with a good long-term prognosis, and there is a reduced risk of kidney failure with PR [15,16]. Here, the IMN remission rate in the TID + group was significantly lower than that in the TID − group. Further subgroup analysis showed no significant difference between the two groups when using GC combined with CYC. However, the remission rate in the TID + group was significantly lower than that in the TID − group when using GCs combined with CNI. This indicates that it is more difficult for patients with IMN with TID to achieve CR after 1 year of treatment. Among the patients with IMN with GC + CNI, the CR rate of those with TID was lower. The percentage of hypertension before initial treatment was 51.67% in all patients (*N* = 155). The percentage of diabetes before initial treatment was 5.67% (*N* = 17) and after immunotherapy was 7.67% (*N* = 23), with no statistically significant difference (*p* > .05). All diabetic patients were given glucose-lowering adjuvant therapy during the treatment period. Blood glucose and blood pressure control were stable, and blood pressure control and diabetes mellitus could basically exclude bias.

Data after one year of follow-up showed that 24-h urine protein and BUN were significantly higher in the TID + group than in the TID − group. In the GC combined with the CNI regimen, 24-h urine protein, Scr, and BUN were significantly higher in the TID + group than in the TID − group. Both CsA and TAC are CNIs, and the mechanism of action of both is competitive binding to calcium-regulated neurophosphatase, which inhibits the activity of the enzyme. It has been reported that excessive doses of cyclosporine can lead to increased kidney vascular resistance, especially in glomerular afferent arterioles, resulting in kidney ischemia. This might be the mechanism responsible for chronic tubular interstitial and vascular changes associated with chronic calcineurin toxicity [17]. Regarding CNI nephrotoxicity, some studies have shown that CNI can damage the glomerulus, kidney tubules, kidney vessels, and other tissue structures. A randomized controlled trial conducted repeated kidney biopsies to verify whether TAC caused kidney injury and found no pathological signs of nephrotoxicity [18]. High TAC concentrations increase the risk of kidney function decline, consistent with our findings. After 1 year of follow-up, there was a significant improvement in the important laboratory parameters after treatment compared to the pretreatment period. Here, patients with IMN with a large amount of proteinuria and normal kidney function were enrolled. After combined GC and immunosuppressant treatment, the CR + PR rate of the patients was high. After active proteinuria reduction treatment, the risk of progression to ESKD was significantly reduced.

The survival curve showed that the kidney survival rate in the TID + group was lower than that in the TID − group. Risk factor analysis showed that 24-h urine protein, β2-microglobulin, anti-PLA2R antibody, chronic kidney tubulointerstitial disease, FSGS, and pathological stage III were risk factors influencing a worse kidney condition. 24-h urine protein is an independent risk factor for the deterioration of kidney function. Ke Zuo et al. found that reduced eGFR and chronic tubulointerstitial injury were independent risk factors for ESKD [19]. Shiiki et al. suggested that tubulointerstitial injury is an important predictor of the progression of nephrotic syndrome to ESKD [20]. Accumulating evidence shows that kidney function decline correlates better with tubulointerstitial damage than with glomerular injury [21–23]. However, some studies have found that tubulointerstitial damage is not an independent risk factor for the progression of kidney function [24–26]. This study found that although IMN with TID was a univariate predictor for the progression of kidney function, it was not an independent risk factor in the multivariate analysis. Due to the features of a single-center retrospective study and short follow-up period, combined with the clinical and pathological findings, we believe that IMN with TID is still an important prognostic factor.

To date, the underlying mechanisms of TID lesions in IMN are unclear. It is believed that excess protein reaching the kidney tubules is ordinarily absorbed by the tubular epithelium. The innate immune characteristics of tubular epithelial cells (TECs) enable them to act as immune responders to a wide range of insults, with the consequent production and release of bioactive mediators that drive interstitial inflammation and fibrosis. Accumulating evidence shows that kidney function decline correlates better with tubulointerstitial damage than with glomerular injury. Furthermore, TECs also play an active role in progressive kidney injury through emerging mechanisms associated with a partial epithelial-mesenchymal transition, cell-cycle arrest at both G1/S and G2/M check points and metabolic disorder [27]. A study showed that the anti-PLA2R antibody level was positively correlated with the urine protein level [28]. Proteinuria leads to the release of proinflammatory cytokines and growth factors. This in turn recruits inflammatory cells to establish tubular interstitial infiltration and trigger fibrosis. Our study showed that 24HURPO levels were significantly higher in IMN patients with TID + than in TID − patients. This may explain why the anti-PLA2R antibody levels were higher in the TID + group. Urinary KIM-1 also positively correlated with the levels of Scr, BUN, and proteinuria (the premise of prognosis and severity) and negatively correlated with eGFR level, suggesting that urinary KIM-1 is an early complementary marker of IMN and closely related to IMN severity [29]. This suggests that the detection of urinary KIM-1 early in the disease may be suggestive of tubulointerstitial damage.

This study had certain limitations. First, this was a single-center, retrospective clinical study with small sample size. Second, the observation time was short, and the number of cases reaching the study’s endpoint was limited. Third, TID was not subdivided or stratified. In the future, we will expand the sample size of the study’s preliminary results and conduct a long-term study on the kidney.

## Conclusion

The levels of 24-h urine protein, BUN, Scr, Cystatin C, anti-PLA2R antibody, and β2-microglobulin in patients with IMN with TID were higher than those in patients without TID, and the pathological damage was more severe. Notably, MN combined with TID is a risk factor for worsening kidney function rather than an independent risk factor.

## Data Availability

The data that support the findings of this study are available from the corresponding author upon reasonable request.
